# A Novel Arch-Shape Nanogenerator Based on Piezoelectric and Triboelectric Mechanism for Mechanical Energy Harvesting

**DOI:** 10.3390/nano5010036

**Published:** 2014-12-26

**Authors:** Chenyang Xue, Junyang Li, Qiang Zhang, Zhibo Zhang, Zhenyin Hai, Libo Gao, Ruiting Feng, Jun Tang, Jun Liu, Wendong Zhang, Dong Sun

**Affiliations:** 1 Key Laboratory of Instrumentation Science and Dynamic Measurement of Ministry of Education, North University of China, Taiyuan 030051, China; E-Mails: junyangli3-c@my.cityu.edu.hk (J.L.); leeyfrank@gmail.com (Q.Z.); markbzhang@gmail.com (Z.Z.); Haizhenyin@gmail.com (Z.H.); nucgaolibo@gmail.com (L.G.); fengruiting401@gmail.com (R.F.); tangjun16@nuc.edu.cn (J.T.); liuj@nuc.edu.cn (J.L.); wdzhang@sxedu.gov.cn (W.Z.); medsun@cityu.edu.hk (D.S.); 2 Science and Technology on Electronic Test and Measurement Laboratory, North University of China, Taiyuan 030051, China; 3 Department of Mechanical and Biomedical Engineering, City University of Hong Kong, Kowloon 999077, Hong Kong

**Keywords:** arch-shape, piezoelectric, triboelectric, mechanical energy

## Abstract

A simple and cost-effective approach was developed to fabricate piezoelectric and triboelectric nanogenerator (P-TENG) with high electrical output. Additionally, pyramid micro structures fabricated atop a polydimethylsiloxane (PDMS) surface were employed to enhance the device performance. Furthermore, piezoelectric barium titanate (BT) nanoparticles and multiwalled carbon nanotube (MWCNT) were mixed in the PDMS film during the forming process. Meanwhile, the composition of the film was optimized to achieve output performance, and favorable toughness was achieved after thermal curing. An arch-shape ITO/PET electrode was attached to the upper side of the polarized composite film and an aluminum film was placed under it as the bottom electrode. With periodic external force at 20 Hz, electrical output of this P-TENG, reached a peak voltage of 22 V and current of 9 μA with a peak current density of 1.13 μA/cm^2^, which was six times that of the triboelectric generator without BT and MWCNT nanoparticles. The nanogenerator can be directly used to lighten 28 commercial light-emitting diodes (LEDs) without any energy storage unit or rectification circuit under human footfalls.

## 1. Introduction

As an attractive substitution for conventional power supply, harvesting energy from the ambient environment is an effective way to achieve self-powered systems and further fulfill large-scale energy needs [1,2,3]. A nanogenerator converting vibrational and mechanical energy to electric energy based on the piezoelectric effect has been proved to be an effective approach by Wang and Song [1] and Lee’s groups [4]. Wang and co-workers have used piezoelectric nanowire arrays to develop nanogenerators, which showed the feasibility to power commercial light-emitting diode (LED) [5], liquid crystal displays [6], and wireless data transmission [7]. BaTiO_3_ (BT) nanoparticles and carbon nanotubes have been utilized to obtain a piezoelectric nanocomposite generator [8]. Subsequently, technology has thus been further introduced to employ a system that can convert frictional energy sources into electrical energy [9,10,11,12,13].

Recently, different configurations that accommodate the needs of harvesting energy that has been deprived from diverse mechanical motions, such as human walking, liquid-wave moving, and wind blowing, were introduced by changing the structure of the nanogenerators. Although piezoelectric cantilevers and structured PDMS film have been combined to enhance the structure performance [14], there have been few reports about the technology of combining piezoelectric and triboelectric mechanism to optimize the material property and thereby obtain high voltage output. 

Here, we developed a hybrid nanogenerator based on piezoelectric and triboelectric principle by employing surface-nanostructured polydimethylsiloxane (PDMS) film, barium titanate (BT) and multiwalled carbon nanotube (MWCNT) nanoparticles. The BT and MWCNT were integrated to PDMS film to increase the output voltage. Doctor-blade coating technology was used to fabricate the device and the output performances were investigated.

## 2. Results and Discussion

### 2.1. Frequency Effect

**Figure 1:**
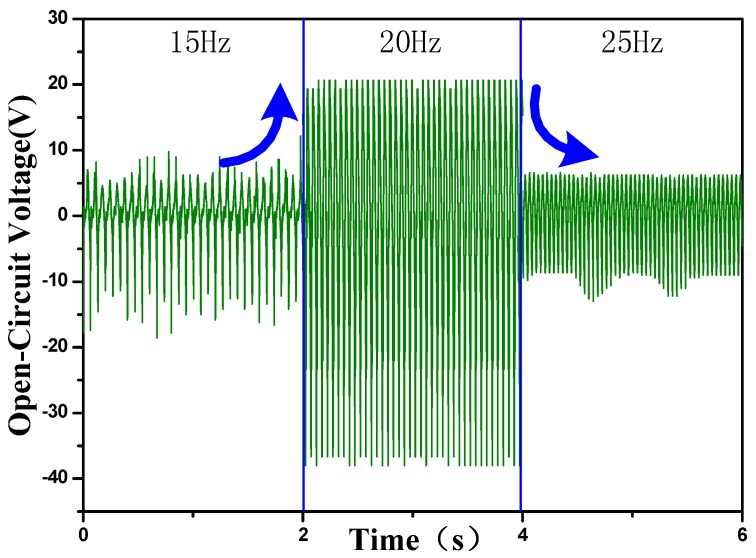
Output performance of the arch-shape piezoelectric and triboelectric nanogenerator (P-TENG) with nanostructured polydimethylsiloxane (PDMS) film under external forces at different frequencies.

Performance of the arch-shape triboelectric nanogenerator (P-TENG) with surface-structured PDMS under the compressive force at different frequencies is shown in Figure 1. As the external force frequency increased from 15 to 20 Hz, the open-circuit voltage increased gradually from 10 to 22 V. Open-circuit voltage was maximized at a frequency of about 20 Hz. However, the open-circuit voltage declines gradually to 5 V at 25 Hz because of the under-releasing of the P-TENG. This is mainly because, when the cycle of the compressive force is too short or the frequency is too high, the nanogenerator cannot recover to the original position before the next force impact [13].

### 2.2. Electrical Output Performance

**Figure 2:**
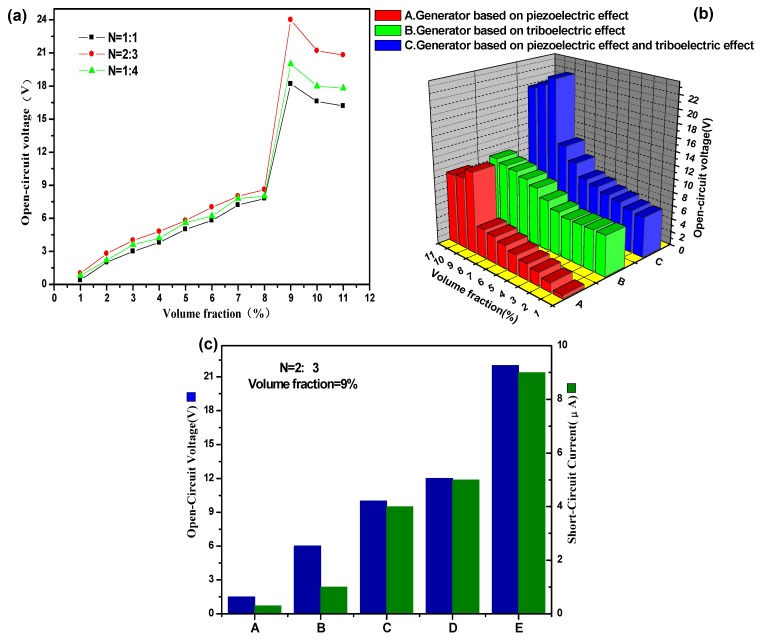
(**a**) Open-circuit voltage of the arch-shaped P-TENG with different *N* at 20 Hz. (**b**) Open-circuit voltage of the P-TENG with different compositions based on *N* = 2:3, including: A. generator based on piezoelectric effect; B. generator based on triboelectric effect; C. Generator based on both the piezoelectric and triboelectric effect; (**c**) Open-circuit voltage and short-circuit current of the arch-shape nanogenerator with different compositions, when the doping radio was fixed (*N* = 2:3, *V* = 9%), including: sample A: flat PDMS film; sample B: PDMS film with only micro pyramid arrays; sample C: PDMS film with micro pyramid arrays, unpolarized barium titanate (BT) nanoparticles and carbon nanotube (CNT); sample D: PDMS with only polarized BT nanoparticles and CNT; sample E: PDMS film with micro pyramid arrays, polarized BT nanoparticles and CNT (P-TENG).

At 20 Hz, open-circuit voltages of the arch-shaped P-TENG with different *N* (The volume ratio of BT to MWCNT) and *V* (the volume percentage of BT and MWCNT mixtures/PDMS matrix) are shown in Figure 2a. More specifically, the output voltage was very low at about 1 V at 1%. Then the voltage increased gradually from *V* = 2% to 9%, peaking at 22 V with *N* = 2:3 and *V* = 9%. After this point, the output voltage declined slowly to about 20 V until *V* = 11%. Hence, *N* = 2:3 and *V* = 9% are the optimal doping ratio, which is further verified in the Figure 2b. From sample A of Figure 2b, it can be seen that the output trend is consistent with that in Figure 2a, showing a constant improvement. Sample B of Figure 2b shows that open-circuit voltage of the generator based on the triboelectric effect was slightly changed at the beginning *(V* = 1% to *V* = 4%) and then it gradually increased after *V* = 5% to the end. This may be because, as the value of *V* increased, some particles inside the composite film could pass through the surface of film, resulting in an increase in the triboelectric effect. Sample C of Figure 2b shows that there was a gradual increase from *V* = 1% to *V* = 8% and then the output voltage witnessed a sharp increase at *V* = 9%, finally it declined slightly. When *V* = 10% or 11%, since the density of particles in the composite film was very high, the piezoelectric particles contacted, thus further weakening the overall output performance. Hence, the P-TENG shown in sample C of Figure 2b displayed superior performance.

The output performances of the arch-shape nanogenerator P-TENG, with certain doping conditions (*N* = 2:3, *V* = 9%), are shown in Figure 2c. In brief, the open-circuit voltage and short-circuit current were enhanced after adding piezoelectric nanoparticles. It has been demonstrated that triboelectric effect can enhance the output performance most effectively [13]. Here, a P-TENG were introduced, which not only increased the effective roughness (compared with sample B of Figure 2c and sample C of Figure 2c but also enhanced the nanogenerator performance by introducing piezoelectric effect. The advantages of the P-TENG had been displayed by comparing the output performances of five samples (A–E) in Figure 2c. More specifically, the PDMS film with micro pyramid arrays, unpolarized BT nanoparticles and CNT in sample C of Figure 2c has enhanced the open-circuit voltage and short-circuit current by 66.7% and 166.7%. Moreover, the novel P-TENG shown in sample E of Figure 2c exhibited more attractive performance, whose voltage and the current were further enhanced by 367% and 500% as compared with in sample B of Figure 2c. Meanwhile, the open-circuit voltage and the short-circuit current were increased by 244% and 240%, as compared with that of the PDMS with polarized BT nanoparticles and CNT in sample D of Figure 2c.

Actually, in the mixed system, granular polarized BT particles may form aggregates and chains. The MWCNTs with great aspect ratio can form a long-conducting channel while fibrous MWCNTs could introduce a bridging effect between the BT-chain or aggregates. Similarly, a BT particles chain or aggregates can be connected to adjacent MWCNTs. Through synergy, a relatively complete network structure in a specific doping ratio was formed and thereby the conductivity of the composite material performance was enhanced [15]. However, the composite output voltage declined slightly after *N* = 2:3, *V* = 9%. This may be because the density of particles in the composite film was increased, resulting in the piezoelectric particles contacting each other directly and further weakening the overall output performance of the film.

### 2.3. Working Principle

**Figure 3:**
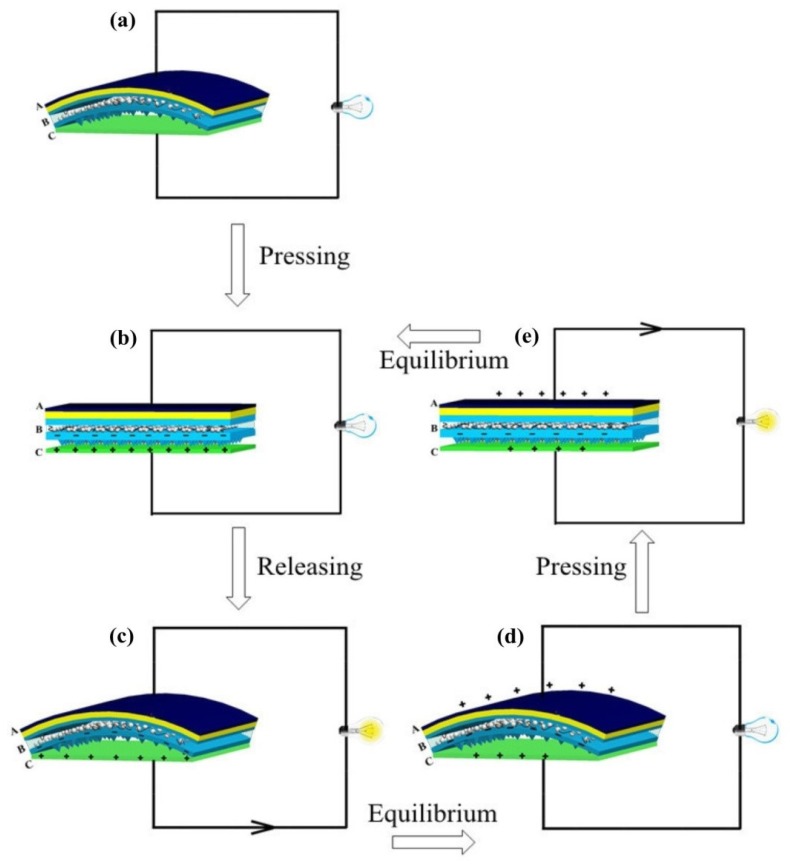
Working principle of this arch-shape nanogenerator. (**a**–**e**) Schematic diagram shows the working principle of the arch-shape P-TENG.

The working principle of this arch-shape nanogenerator can be depicted as followed in Figure 3, combining piezoelectric and triboelectric mechanism. The operation of P-TENG is realized by applying a cycled compressive force onto the whole area of the device, so that the bending plates will be periodically pressed to contact closely with each other. In order to simplify the description, the upper surface of ITO/BET film is labeled as layer A and the surface of polarizing film is marked as layer B while the lower surface of the Al foil is labeled as layer C.

The distribution of triboelectric charge (σ_T_) and piezoelectric charge (σ_P_) of A, B, C three layers in one pressing cycle was shown in Table 1. At the origin state, the polarized piezoelectric film is in the bending state, so layer A induces negative piezoelectric charge –σ_P_, but there is no friction effect and no charge transferred as well, thus, no electric potential. In the pressing process, the charge –σ_P_ still exists in layer A and electrons will be injected from Al to the PDMS surface because of friction effect, leaving positive triboelectric charges +σ_T_ and piezoelectric charge +σ_P_ on the layer C while the negative triboelectric charges will be preserved on the layer B surface due to the character of the insulator [16]. Such a charge transfer process will continue in the initial few hundreds of cycles until the accumulated charges reach saturation and it is depicted in the part (b) of Table 1. When the pressing force disappears, the P-TENG will return to its original arch shape immediately so that an air gap will form again between the layer B and layer C, resulting in a much higher potential on the layer C than layer B [10]. As it is illustrated in Figure 3c, this potential difference will drive the stream of positive charges from layer C to layer A through the external load until the potential difference is fully counteracted by the transferred charges (Δσ). This will give rise to the layer A with a surface charge density of +Δσ − σ_P_ and the layer C is left with +σ_T_ − Δσ as shown in Figure 3d. Soon after, when the P-TENG is pressed again to let the layers B and C contact, these redistributed charges will conversely establish a positive potential on layer A in Figure 3e, which will drive the Δσ to flow back to the layer C. In this way, a period is achieved and the device will return to the equilibrium state in Figure 3b. This is a whole cycle of electricity generation based on piezoelectric and triboelectric mechanism. Therefore, we can see from Table 1 that when a friction effect exists on its own, the charge transfer of this structure is Δσ while the charge transfer is σ_P_ for piezoelectric effect only through combining piezoelectric effect and triboelectric effect. We can easily establish that the charge transfer of arch-shape structure is σ_P_ + Δσ.

**Table 1:** The distribution of triboelectric charge (σ_T_) and piezoelectric charge (σ_P_) of A, B, C three layers in one pressing cycle. Δσ: transferred charges.

Layer	(a)		(b)		(c)		(d)		(e)	
	piezo	tribo	piezo	tribo	piezo	tribo	piezo	tribo	piezo	tribo
A	−σ_P_	0	−σ_P_	0	−σ_P_	0	−σ_P_	+Δ_σ_	−σ_p_	+Δ_σ_
B	0	0	0	−σ_T_	0	−σ_T_	0	−σ_T_	0	−σ_T_
C	0	0	+σ_P_	+σ_T_	+σ_P_	+σ_T_	0	+σ_T_ − Δσ	0	+σ_T_ − Δσ

### 2.4. Application

**Figure 4:**
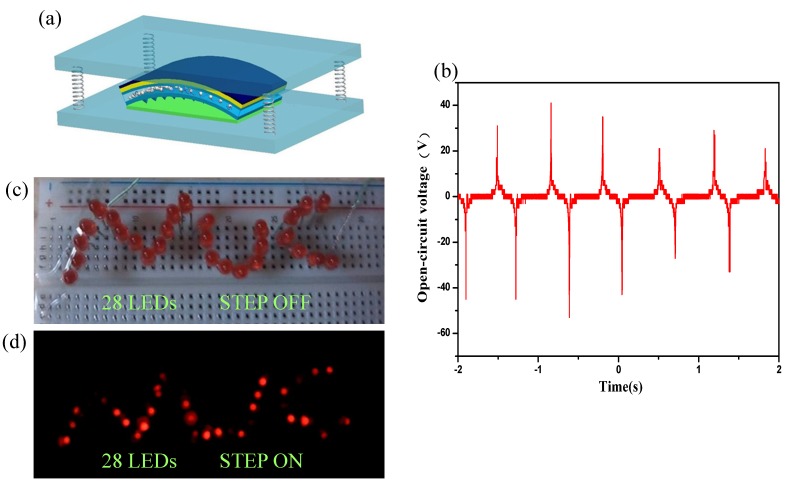
Applications of the arch-shape P-TENG. (**a**) Diagram of the P-TENG between two plexiglasses with springs; (**b**) The output open circuit voltage is about 30 V under footstep. (**c**,**d**) When footstep falls on the P-TENG, 28 paralleled commercial LEDs were lightened without using any energy storage device or rectification circuit. Notes: All LEDs are connected in serial.

The output performance of this P-TENG has a highest peak voltage of 22 V and current of 9 μA (The output was measured using an oscilloscope with a 10 MΩ probe, and the output current was measured by a low-value series resistance of 9 kΩ). This output is large enough to power many micro/nanosystems, even some commercial electronic devices. Figure 4 shows that the P-TENG was capable of lighting up as many as 28 commercial LED bulbs simultaneously, by converting commonly ambient mechanical energy such as human footfalls.

## 3. Experimental

### 3.1. Reagents and Apparatus

BaTiO_3_ nanoparticles (BT, average particle diameter of 30 nm) and multiwalled carbon nanotubes (MWCNT, average particle diameter of 20 nm) were provided by DK nano technology Co. Ltd. (Beijing, China) and PDMS (Sylgard 184, Dow Corning) was purchased from Zhenkonghang adhesive technology Co. Ltd. (Shenzhen, China). Pure alcohol was supplied by Sinopharm Chemical Reagent Co. Ltd. (Shanghai, China). ITO/PET was purchased from Kaiwei Electronic Components Co. Ltd. (Zhuhai, China). The thickness of the composite film was controlled by using a doctor-blade coating machine (AC-150, Hefei, China) and then cured in a vacuum oven (ZK-1S type, Changzhou, China). With a JEOL JEM-1200EX transmission electron microscope (Tokyo, Japan) and S4700 scanning electron microscope (Tokyo, Japan), TEM and scanning electron microscope (SEM) measurements were performed. Open-circuit voltage and short-circuit current of the arch-shape P-TENG were measured with a digital oscilloscope (Tektronix DPO 2024 type, Beaverto, OR, USA). A vibration system, including a waveform generator (RIGOL DG1022, Beijing, China), a power amplifier (SINOCERA YE5871A, Shanghai, China) and a vibration platform (SINOCERA, Shanghai, China), was assembled to apply a cycled compressive force with controllable frequency.

### 3.2. Preparation of the Composites

**Figure 5:**
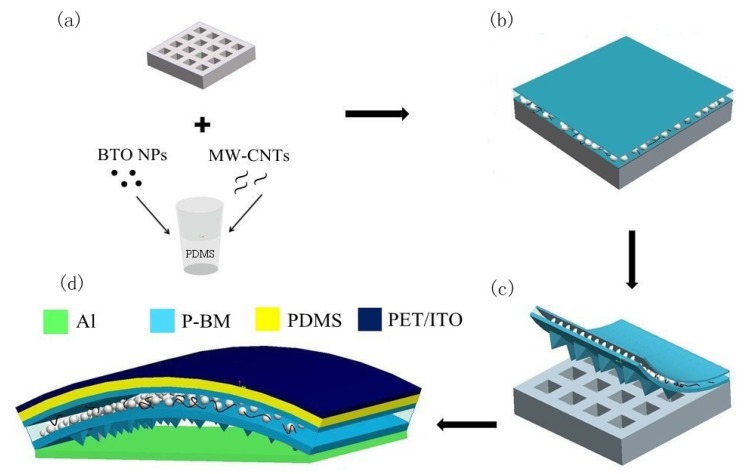
(**a**–**d**) Schematic view of the structural design and fabrication process flowchart of the P-TENG device (the total size of the P-BM composite films is 2 cm × 4 cm × 0.3 mm).

Figure 5 shows the schematic view of structural design and fabrication process flowchart of the P-TENG. In this work, we fabricated pyramids on the polymer surfaces to increase the triboelectric power output. To make patterned PDMS films, Si wafer molds were first fabricated by using traditional photolithography method, following by dry or wet etching process to achieve pyramids. BaTiO_3_ NPs were mixed with MWCNTs in specific volume proportions including *N* = 1:1, *N* = 2:3 and *N* = 1:4. The mixtures were then stirred for approximately 5 h in ethanol with a magnetic stirrer. Liquid PDMS elastomer and cross-linker were mixed and degassed to achieve quantitative PDMS matrix (Figure 5a). After the subsequent drying and granulation, the well-mixed nanomaterials with three different proportions were poured into PDMS matrix, with the volume ratios of mixtures/PDMS matrix at *V* = 1% to *V* = 11%. Then, 33 P-BM composite films (PDMS mixtured with BaTiO_3_ NPs and MWCNTs) were obtained. The composite films were polarized at 3.6 kV/mm, and 90 °C for 60 min.

**Figure 6:**
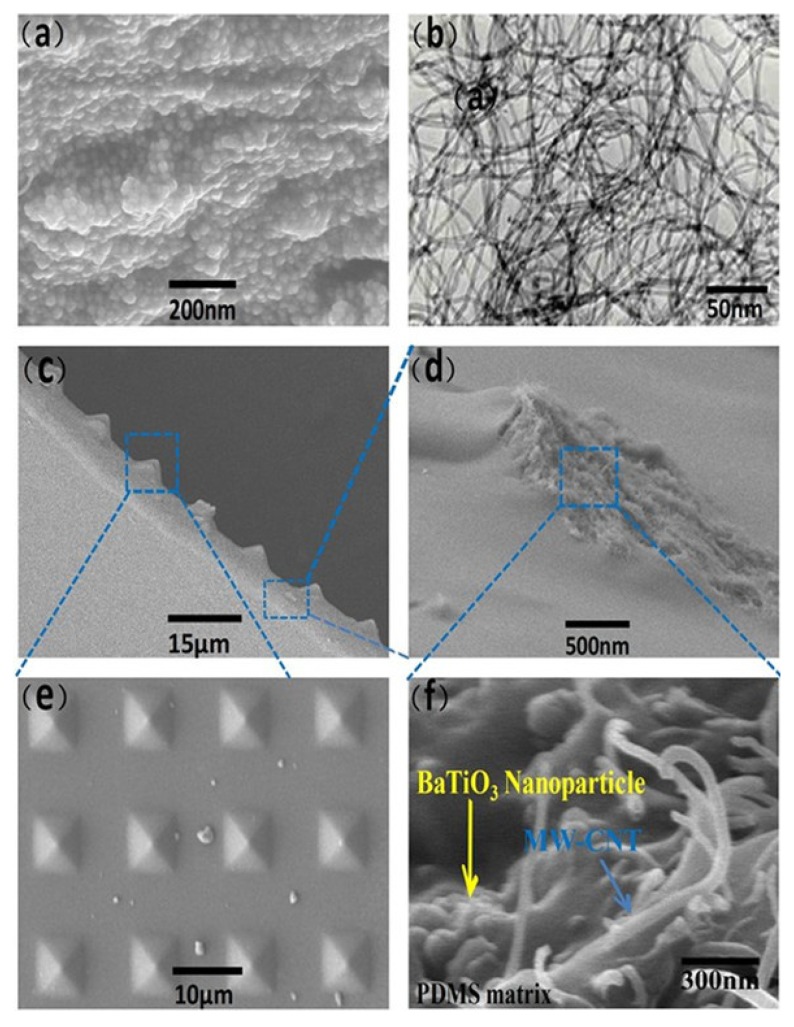
Structure characterization of the P-BM composite films. (**a**) SEM image of the BaTiO_3_ NPs; (**b**) TEM image of multiwalled carbon nanotube (MWCNTs) with a diameter of 20 nm and a length of 20 μm; (**c**) SEM image of the pyramid PDMS thin film with BaTiO_3_ NPs and MWCNTs; (**d**) Enlarge photograph of the PDMS thin film tore locally by tweezers; (**e**) High-magnification SEM image of the patterned PDMS surfaces with pyramids features; (**f**) The high-magnification SEM image of mixing effect about BaTiO_3_ NPs and MWCNTs.

Then, the surface of the molds was treated with trimethylchlorosilane to prevent the PDMS film from sticking to the molds, while a doctor-blade coating machine was utilized to enable the P-BM samples to be on the surface of the mold (Figure 5b). After thermal curing, a uniform PDMS layer doped with piezoelectric materials was peeled off, with the inverse of the original pattern features on the surface of the mold, with a thickness of 300 μm (Figure 5c). Then the piezoelectric thin film was placed closely in two aluminum foil pieces and put in a polarization set-up to be polarized. Subsequently, the unstructured side of the composite film was fixed on the insulation surface of a clean baked ITO/PET membrane (uniformly baked by soldering irons for several seconds to form the optimal planar separation distance between the top and bottom surface [16]) by a thin PDMS bonding layer, and then the aluminum foil was used as a bottom electrode to form an arch-shape device (Figure 5d). A detailed fabrication protocol and typical structure of the nanogenerator are schematically shown in Figure 4. Figure 6 shows SEM images of the arch-shape P-TENG. From Figure 6e, it can be seen that there are some microparticles on surface of the P-BM films besides the pyramid micro structures, which may improve the surface friction effect further. The entire preparation process of the device is simple and low-cost, making it possible for large-scale production and practical applications.

## 4. Conclusions

A P-TENG based on the piezoelectric-triboelectric process was demonstrated and it can be used as an energy harvester for human footfalls. This design can significantly improve the output efficiency of the nanogenerator by combining the piezoelectric and triboelectric effect. For a typical P-TENG with polarized BT and MWCNT nanoparticles, the electrical output achieved a peak voltage of 22 V and current of 9 μA with a peak current density of 1.13 μA/cm^2^, which is six times as high as that of the generator without BT and MWCNT nanoparticles. Owing to the simple and low-cost production process, the P-TENG exhibits great advantages in industrial production and practical applications as well as manufacturability and capability of integration with other processing technologies. Finally, with such a power output, we envision future energy harvesting by this P-TENG not only from human footfall but also from rolling wheels, wind power and ocean waves.
